# Elevated DHODH expression promotes cell proliferation via stabilizing β-catenin in esophageal squamous cell carcinoma

**DOI:** 10.1038/s41419-020-03044-1

**Published:** 2020-10-15

**Authors:** Yu Qian, Xiao Liang, Pengzhou Kong, Yikun Cheng, Heyang Cui, Ting Yan, Jinghao Wang, Ling Zhang, Yiqian Liu, Shiping Guo, Xiaolong Cheng, Yongping Cui

**Affiliations:** 1 Cancer Institute, Peking University Shenzhen Hospital, Shenzhen Peking University-Hong Kong University of Science and Technology (PKU-HKUST) Medical Center, 518035 Shenzhen, People’s Republic of China; 2 Department of Pathology & Shanxi Key Laboratory of Carcinogenesis and Translational Research on Esophageal Cancer, Shanxi Medical University, 030001 Taiyuan, Shanxi People’s Republic of China; 3 Department of Obstetrics & Gynecology, the Second Hospital, Shanxi Medical University, Taiyuan, Shanxi China; 4 Department of Tumor Surgery, Shanxi Cancer Hospital, Taiyuan, Shanxi China

**Keywords:** Oesophageal cancer, Prognostic markers

## Abstract

As a key enzyme in de novo pyrimidine biosynthesis, the expression level of dihydroorotate dehydrogenase (DHODH) has been reported to be elevated in various types of malignant tumors and its tumor-promoting effect was considered to relate to its pyrimidine synthesis function. Here, we revealed one intriguing potential mechanism that DHODH modulated β-catenin signaling in esophageal squamous cell carcinoma (ESCC). We demonstrated that DHODH directly bound to the NH2 terminal of β-catenin, thereby, interrupting the interaction of GSK3β with β-catenin and leading to the abrogation of β-catenin degradation and accumulation of β-catenin in the nucleus, which in turn, resulted in the activation of β-catenin downstream genes, including *CCND1*, *E2F3*, *Nanog*, and *OCT4*. We further demonstrated that the regulation of β-catenin by DHODH was independent of DHODH catalyzing activity. Univariate and multivariate analyses suggested that DHODH expression might be an independent prognostic factor for ESCC patients. Collectively, our study highlights the pivotal role of DHODH mediated β-catenin signaling and indicates that DHODH may act as a multi-functional switcher from catalyzing pyrimidine metabolism to regulating tumor-related signaling pathways in ESCC.

## Introduction

Esophageal squamous cell carcinoma (ESCC) is the ninth leading cause of cancer-related death worldwide, with over 515,000 new cases annually^1–3^. China has the highest incidence and mortality rate of ESCC; ~70% of ESCC occur in China^4,5^. The 5-year survival rate of ESCC tends to be poor due to the lacking of early diagnosis, limited therapeutic options and frequent relapse^6,7^. Therefore, efforts are needed to explore the molecular mechanism underlying ESCC tumorigenesis for identification of molecular markers for diagnosing or predicting patient outcomes.

Dihydroorotate dehydrogenase (DHODH) is the rate-limiting enzyme in the uridine monophosphate (UMP) biosynthetic pathway and mostly located in the inner membrane of mitochondria, catalyzing the transformation of dihydroorotate to orotate dependent of Mg^2+^ and ATP^8,9^. Mutations of *DHODH* have been associated with various genetic diseases. For example, DHODH G202A, R346W causes deficient protein stability and R135C impairs the enzymatic activity, which are linked to Miller syndrome^10,11^. *DHODH* polymorphism was reported to be linked with rheumatoid arthritis and lung cancer as well^12–14^. Additionally, enhanced activity of DHODH has been implicated as a biomarker of malignant tumor including gastric cancer and skin cancer^15,16^. Ultraviolet radiation could transcriptionally induce DHODH expression by activating STAT3^17^. Inhibition of DHODH leads to the starvation of pyrimidine pool and thereby decreases DNA and RNA synthesis and cell proliferation^18,19^. Meanwhile, DHODH has impacts on mitochondrial Oxidative phosphorylation (OXPHOS), which is also responsible for de novo pyrimidine synthesis pathway^20^. However, little is known about the biological function of *DHODH* and molecular mechanism responsible for ESCC.

In this study, we report that DHODH may directly interact with β-catenin and mediate β-catenin stabilization by interpreting its phosphorylation, thus prevent the polyubiquitination and induce β-catenin nuclear translocation, leading to ESCC cells proliferation and tumorigenesis. Furthermore, we demonstrated that DHODH was upregulated in ESCC samples compared with adjacent normal tissues and high expression of DHODH was significantly associated with early stages and shorter patient survival in ESCC.

## Materials and methods

### Clinical samples

This study was approved by the Ethics Committee of Shanxi Medical University (Approval No.2017LL108). The written informed consents were received from all participates. ESCC Tissue microarrays (TMAs) were obtained from Shanghai Outdo Biotechnology Company Ltd. One TMA includes 38 cases of ESCC, 7 cases of carcinoma in situ, 22 cases of atypical hyperplasia, and 10 cases of adjacent non-cancer tissues. The other TMA contains 208 cases of ESCC tissues and non-cancer tissues with clinicopathological parameters (male 162 cases, female 46 cases, age 45–92 years, median age 67 years, 2 to 9-year follow-up).

### Cell lines, reagents, and antibodies

ESCC cell lines KYSE140, KYSE150, KYSE180, KYSE410, KYSE510, KYSE450, KYSE680, ECA109 and immortalized esophageal epithelial cell SHEE were generous gifts from Dr. Qimin Zhan (Key laboratory of Carcinogenesis and Translational Research, Peking University Cancer Hospital & Institute) and stored at Shanxi Key Laboratory of Carcinogenesis and Translational Research on Esophageal Cancer, Shanxi Medical University. Cells were authenticated and tested as mycoplasma free. Cells were grown in RPMI1640 media (Hyclone, USA) supplementary with 10% fetal bovine serum (FBS), 100 U/ml penicillin, and 100 μg/ml streptomycin.

Detailed reagents and antibodies used in this study were described in the Supplementary Table 1. The lentiviruses encoding two different short-hairpin RNA (shRNA) sequences specific against DHODH (sequence from Sigma-Aldrich) were purchased from Cyagen Biosciences Inc. After confirming the knocking down efficiency, two shRNAs were used as a pool. DHODH cDNA was cloned into pcDNA3.1 by BamH I and Xba I using primers as shown in Supplementary Table 2.

### Animal experiments

Mice study was proceeded according to the guide for the Care and Use of Laboratory Animals in Shanxi Medical University. Mouse xenograft assay was performed on 4- to 6-week-old male BALB/c nude mice, and each group has 6 mice according to the guidelines. In all, 3 × 10^6^ DHODH-overexpressing KYSE150 cells or DHODH knocking down ECA109 cells, were subcutaneously injected into the left oxter of nude mice, respectively. KYSE150 cells or ECA109 cells were used as control, respectively. After five weeks, mice were sacrificed and tumors were removed and measured. Tumor volumes were measured using caliper and calculated by the formula: length (mm) × width (mm)^2^/2.

### Immunohistochemical staining (IHC)

TMAs and serial 4 μm paraffin-embedded sections from each xenograft tumor were subjected to IHC staining to evaluate the expression level of specific proteins. Briefly, the slides were deparaffinized in xylene, rehydrated in graded ethanol and treated with 3% H_2_O_2_ to block endogenous peroxidase. Then antigen retrieval was performed in citrate buffer pH 6.0 for 3 min. After three washes with phosphate buffer saline (PBS), slides were incubated with primary antibodies at 4 °C overnight. After washing with PBS, the slides were incubated with MaxVision Mouse/Rabbit (MXB biotechnology, KIT-5020) and visualized with DAB and counterstained with hematoxylin. All images were captured by Aperio Scan Scope (AperioTechnology Inc, USA). Protein expression levels were analyzed using Aperio Cytoplasma 2.0 and Nuclear v.9 software.

### Cell proliferation assay

Cell proliferation was measured by CCK-8 assay and colony formation assay. For CCK-8 assay, 1500 indicated cells were seeded into 96-well plates. Ten microliters of CCK-8 solution (Boster, China) was added to each well and incubated at 37 °C for 1 h. The absorbance value was measured at 450 nm. For colony formation assay, 2000 indicated cells were seeded into 6-well plates and cultured for 2 weeks and stained with 0.1% crystal violet.

### Flow cytometry

For apoptosis analysis, cells were harvested, washed with PBS and binding buffer, then stained with Annexin V-FITC and PI using Annexin V-FITC/PI double staining kit (KeyGen Biotech) for 30 min. Apoptotic cells were detected by flow cytometry using BD Calibur. For cell cycle, cells were suspended in pre-cold 70% ethanol, then washed with PBS, incubated with RNase A, and stained with PI using Cell cycle detection kit (KeyGen Biotech). Cell cycle proportion was measured using BD Calibur.

### Immunofluorescence

Cells were cultured on the coverslips and stained with MitoTracker DeepRed FM (Cellsignaling Technology) to indicate mitochondria according to the manufacture’s protocol. Then, cells were permeabilized with 0.25%Triton X-100/PBS. Post 30 min, cells were incubated with 0.5% BSA for 1 h and primary antibodies overnight. Then, cells were washed and incubated with Alexa Fluor-labeled secondary antibodies (Invitrogen). The nuclear were counterstained using DAPI. High-resolution images were captured using the Olympus Fluoview 1000 microscope.

### RNA extraction and quantitative real-time PCR

Total RNA was extracted using RNAiso Kit (Takara) and first-strand cDNA was synthesized using PrimeScript RT Master Kit according to the protocol. Real-time PCR was performed by SYBR Green methods (Takara) on StepOne plus real-time PCR system (Applied Biosystem). The gene expression was measured by −2^ΔΔCT^. β-actin was used as internal control. The primers used were shown in Supplementary Table 2.

### RNA-sequencing

The *DHODH* knockdown KYSE150, KYSE180, ECA109 cells and matched control cells were subjected to RNA sequencing. Total RNA extraction and sequencing were performed by Annoroad Gene Technology (Beijing, China) with standard procedures. Briefly, total RNA was enriched by magnetic oligo-d(T) and fragmented, followed by reverse transcription with random hexamers. Then the secondary cDNA strain was synthesized and purified. After adapter ligation, the library was sequenced using Illumina Hiseq platform with the sequencing strategy PE150. Data analysis was performed by the TopHat-Cufflinks pipeline, and gene expression values were presented by FPKM (fragment per kilobase of transcript sequence per million mapped reads). Differential gene expressions were analyzed by DEGSeq method with Log2 Ratio ≥ 1, *q* < 0.05 as cutoff value. The differential genes were clustered by gene set enrichment analysis according to Kyoto Encyclopedia of Genes and Genomes (KEGG) database.

### Luciferase reporter assay

TOP/FOP plasmids were generous gift from Dr. Canhua Huang (Sichuan University). Cells were co-transfected with TOP flash/FOP flash luciferase report plasmid, pTK-RL plasmid and DHODH plasmid or empty control for overexpression analysis while sh-DHODH and control shRNA for knockdown analysis using Lipofectamine 2000 reagent. 48 h post transfection, cells were collected. The Top flash and Fop flash luciferase values were measured by TransDetect Double-Luciferase Reporter Assay Kit (TransGene, China) according to the manufacturer’s protocol and normalized by Renilla luciferase activity.

### Western blot and immunoprecipitation (IP)

Cells were lysed in RIPA buffer with protease inhibitor and phosphatase inhibitor cocktails (Roche). Total protein concentration was determined by BCA method. Sixty micrograms of total proteins were loaded into 10% sodium dodecyl sulfate polyacrylamide gel electrophoresis and then transferred onto polyvinylidene fluoride membranes (Millipore). The membranes were incubated with primary antibodies at 4 °C overnight. After washing with TBST, the membranes were incubated with IR-labeled secondary antibodies followed by visualizing using Licor Odyssey CLx. The relative protein quantification was performed by calculating the band intensity in ImageJ. For IP assay, cell lysates were incubated with antibodies overnight and Protein G Plus/Protein A agarose (Millipore) was added for 2 h. The precipitation was boiled with SDS-loading buffer and analyzed by western bolt.

### Glutathione S-transferase (GST) pull-down

Recombinant GST-tagged full-length DHODH and its truncated mutants containing an N-terminal were expressed in *E. coli* BL21 (DE3) and induced with 0.1 mM isopropyl β-d-1-thiogalactopyranoside (IPTG) for 16 h. The bacteria were lysed by ultrasonic (300 W, 8 s treatment, 8 s break for 20 min) and debris was removed by centrifugation. The proteins were harvested by incubating with GST binding agarose beads at 4 °C for 2 h. GST-pull down was performed by the total cell lysis incubation at 4 °C overnight and washing with 50 mM Glutathione at 4 °C for 2 h, the supernatant was boiled with SDS-loading buffer and detected by western blot.

### Bio-layer interferometry binding assay

The bio-layer interferometry assay was performed at 25 °C on the Octet Red96 system (ForteBio) with 50 mM Tris-HCL pH 8.0 as running buffer. Purified GST-DHODH proteins were loaded onto anti-GST coated biosensors for 5 min and the biosensors were equilibrated in the running buffer for 2 min to acquire the baseline. Then, the biosensors were incubated with various concentration of Flag-β-catenin proteins for 5 min, followed by 5 min of dissociation. The data were analyzed and the binding parameters were calculated by software.

### Statistical analysis

All statistical analyses were performed using the SPSS 21.0 software and graphs were visualized using the Prism 5 GraphPad. Data were presented as mean ± SEM from at least three independent experiments. Differences between groups were measured by Student’s *t*-test and ANOVA test. The expressions of DHODH in human tissues were compared by Wilcoxon signed-rank test. The correlation between DHODH level and clinicopathological features was analyzed by Chi-square test. Survival curves were constructed using the Kaplan–Meier method and differences in survival were evaluated using the log-rank test. Univariate and multivariate survival analyses were performed by a Cox proportional hazards regression model. The correlation between DHODH and β-catenin was measured by Pearson correlation coefficients. A *P-*value < 0.05 was considered statistically significant.

## Results

### High expression of DHODH was associated with poor prognosis of ESCC patients

**Fig. 1:**
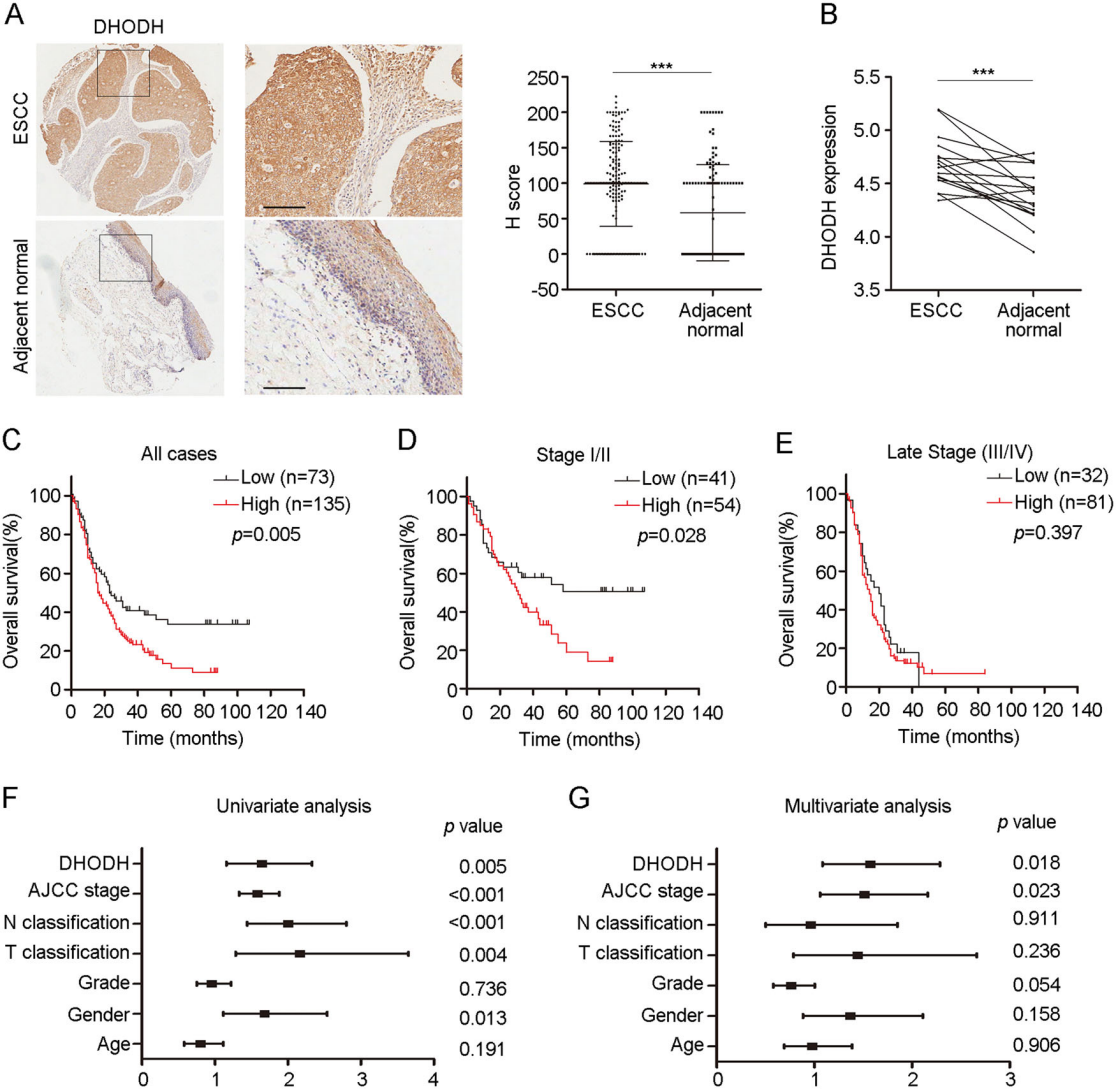
DHODH overexpression is correlated with poor prognosis in ESCC. **a** The protein expression of DHODH was significantly higher in ESCC tissues compared with that in adjacent non-cancer tissues by IHC (left) and the scoring of IHC in 208 cases of ESCC (right). **b** mRNA expression of *DHODH* according to Hu’s cohort. **c** Kaplan–Meier survival analysis of overall survival in 208 cases of ESCC patients. The receiver operating characteristic curve was used to determine the cutoff value. **d**–**e** Kaplan–Meier survival analysis of overall survival in stage I/II patients (**d**) and stage III/IV patients (**e**). The univariate (**f**) and multivariate (**g**) cox regression analysis of DHODH expression and clinicopathological features of ESCC patients. ^***^P < 0.001.

We performed IHC staining in TMAs containing tumor samples and matched normal adjacent tissues from 208 ESCC patients and found a predominant expression of DHODH protein in cytoplasm. DHODH level was significantly higher in tumors compared to that in adjacent normal tissues (Fig. 1a). We also found an elevation of DHODH mRNA in tumors according to Hu’s cohort (Fig. 1b)^21^. Consistently, we observed a marked relative increase of DHODH expression in atypical hyperplasia compared with that of normal tissues, indicating the overexpression of DHODH may play an important role in the initiation of stage of ESCC (Supplementary Fig. S1A, B).

In addition, high DHODH expression was statistically associated with advanced stage (Table 1). In particular, we noticed that patients with high DHODH expression exhibited a worse prognosis (Fig. 1c). However, we found that DHODH expression was correlated with the poor survival of early stage (I and II) patients rather than late stage (III and IV) patients (Fig. 1d–e). Moreover, univariate Cox regression analyses showed that high DHODH expression, gender, T classification, N classification, and AJCC stage was associated with unfavorable overall survival, respectively. Further multivariate Cox regression analysis confirmed the AJCC stage and DHODH expression as potential independent prognostic factors (Fig. 1f–g and Table 2). Collectively, these results suggest that *DHODH* may be a potential predictor to estimate patients’ survival.

**Table 1:** Associations between DHODH expression and clinicopathological parameters of ESCC patients.

Parameters	Case number	DHODH expression		*χ*^2^	*P*
		Low	High		
Overall	208	73	135		
Gender					
Male	162	54	108	0.68	0.41
Female	46	19	27		
Age (years)					
≤60	70	25	45	0.03	0.853
>60	135	45	90		
Unknown	3	3	0		
Tumor differentiation					
Well	48	22	26	4.26	0.119
Moderate	120	41	79		
Poor	40	10	30		
T classification					
T1-2	29	14	15	1.94	0.164
T3-4	176	58	118		
Unkown	3	1	2		
N classification					
N0	90	36	54	1.32	0.251
N1-2	118	37	81		
AJCC stage					
I and II	95	41	54	4.36	0.037*
III and IV	113	32	81		
Status					
Living	52	28	24	9.63	0.002*
Death	156	45	111		

*P < 0.05

**Table 2:** Univariate and multivariate analysis for overall survival.

Parameters	Univariate analysis			Multivariate analysis		
	HR	95% CI	*P*	HR	95% CI	*P*
Age (≤60 vs. >60)	0.802	0.577–1.116	0.191	0.979	0.692–1.386	0.906
Gender (female vs. male)	1.681	1.114–2.538	0.013*	1.371	0.885–2.122	0.158
Histological differentiation (well vs. median vs. poor)	0.959	0.752–1.223	0.736	0.762	0.579–1.005	0.054
T classification (T1 + 2 vs. T3 + 4)	2.165	1.284–3.651	0.004*	1.446	0.785–2.664	0.236
N classification (N0 vs. N1 + 2)	2.01	1.442–2.802	<0.001*	0.963	0.502–1.850	0.911
AJCC stage (I + II vs. III + IV)	1.583	1.331–1.882	<0.001*	1.513	1.058–2.162	0.023*
DHODH expression (low vs. high)	1.644	1.158–2.333	0.005*	1.573	1.083–2.287	0.018*

*P < 0.05

### DHODH promotes cell proliferation and tumor growth in ESCC

**Fig. 2:**
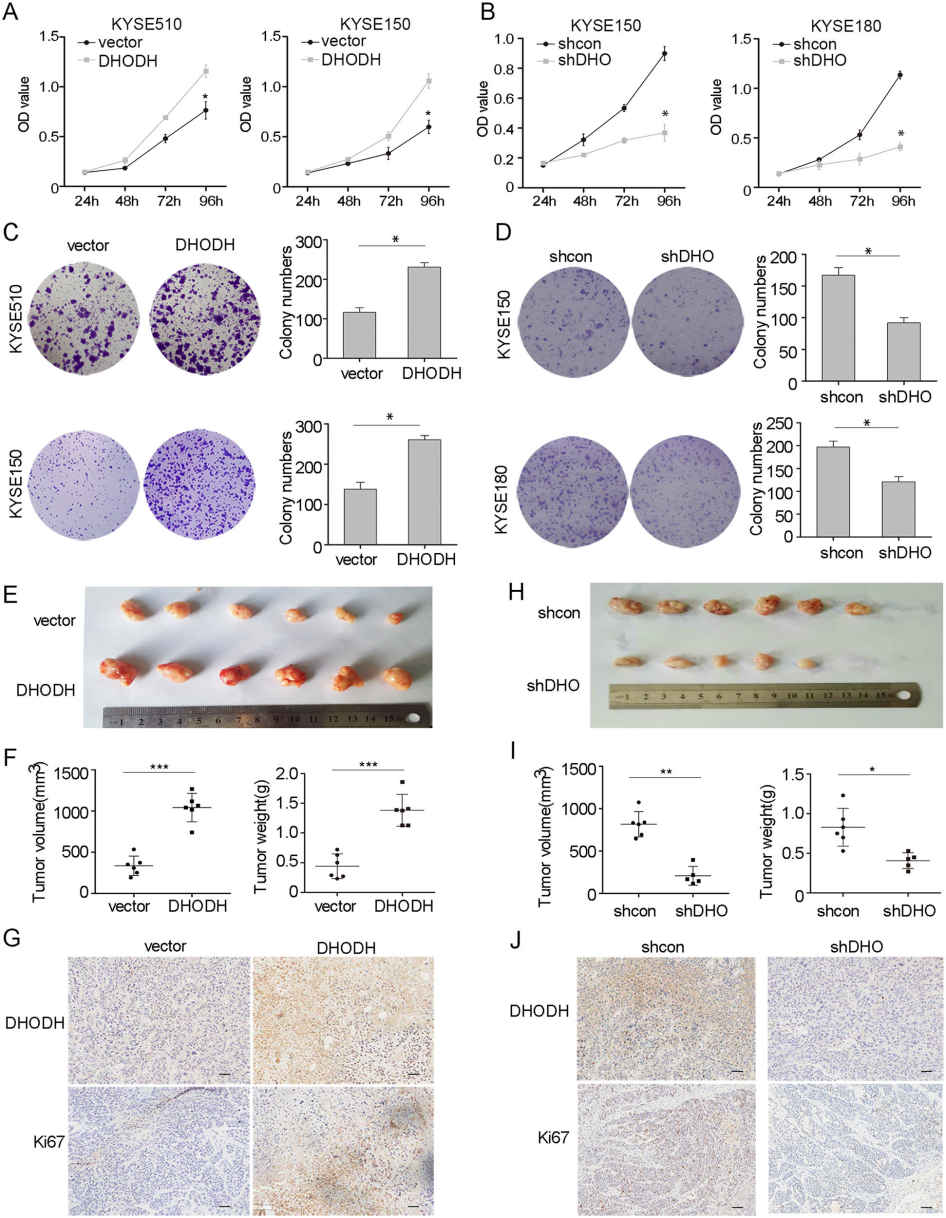
DHODH induces tumorigenesis. **a**, **b** Cell proliferation assay was performed in DHODH overexpressed cells (KYSE510 and KYSE150, **a**), knocked down cells (KYSE150 and KYSE180, **b**), and correspondence control cells, respectively. **c**, **d** Colony formation assay was performed in DHODH overexpressed cells (**c**), DHODH knocked down cells (shDHO), and correspondence control cells (vector or shcon) (**d**). **e** Ectopic expressed DHODH in KYSE150 cells promoted tumor growth in xenograft mice model. **f** The volume and weight of tumors derived from DHODH ectopic expressed cells. **g** The expressions of DHODH and Ki67 were examined in tumor sections. **h** DHODH knockdown suppressed tumor growth in ECA109 cells. **i** The volume and weight of tumors derived from *DHODH* knocked down cells. **j** The expression of DHODH and Ki67 were examined (Bar, 100 μm). Data were representative of three independent experiments and are presented as mean ± SEM. ^*^P < 0.05, ^**^P < 0.01, ^***^P < 0.001.

To discover the biological function of *DHODH* in ESCC, we detected the endogenous expression of DHODH in multiple ESCC cell lines (Supplementary Fig. S1C). We established stably DHODH-overexpressing KYSE510 and KYSE150 cell lines, and knockdown KYSE150, KYSE180 and ECA109 cell lines, respectively (Supplementary Fig. S1D, E). We found that DHODH significantly promoted cell proliferation as shown by CCK-8 assay (Fig. 2a–b) and colony formation assay (Fig. 2c–d). Additionally, leflunomide (Lef), a DHODH inhibitor, exerted a suppressive effect on cell proliferation as well (Supplementary Fig. S1F). Next, we turned to a xenograft mouse model and observed that mice baring DHODH-expressing KYSE150 cells formed malignant tumors more rapidly than that of control cells (Fig. 2e–f*)*, that was confirmed by Ki67 staining assay (Fig. 2g). On the contrary, efficient DHODH deletion significantly prevented tumor growth (Fig. 2h–j). Altogether, these data suggest that DHODH may act as an oncogene via promoting cell proliferation and tumor growth in ESCC.

### Inhibition of DHODH impedes cell cycle and induces apoptosis in ESCC cells

Then we explored the effect of DHODH on cell cycle and apoptosis. Importantly, DHODH knockdown arrested cells in S phase and prolonged cell cycle as monitored by Live cell imaging (Supplementary Fig. S2A, B). Also, we found a remarkable decrease of cyclin A and cyclin B expression after DHODH knocking down (Supplementary Fig. 2C). DHODH knocking down induced apoptosis (Supplementary Fig. S2D). Meanwhile, overexpression of DHODH resulted in the opposite trends. We observed that DHODH inhibited apoptosis and promoted cell cycle (Supplementary Fig. S2E, F). By western blot, we analyzed the expression of pro-apoptosis marker BAX, anti-apoptosis marker BCL2, and cell cycle markers such as E2F3, p53, and p21. Consistently, knockdown of DHODH increased BAX, p53 and p21 expression, which linked with increased apoptosis and cell cycle arrest. On the opposite, DHODH overexpression upregulated BCL2 and cell cycle related genes (Supplementary Fig. S2G, H). Hence, DHODH may play critical roles in the promotion of S-G2/M transition and the regulation of cell apoptosis.

### DHODH enhances Wnt/β-catenin pathway in ESCC

**Fig. 3:**
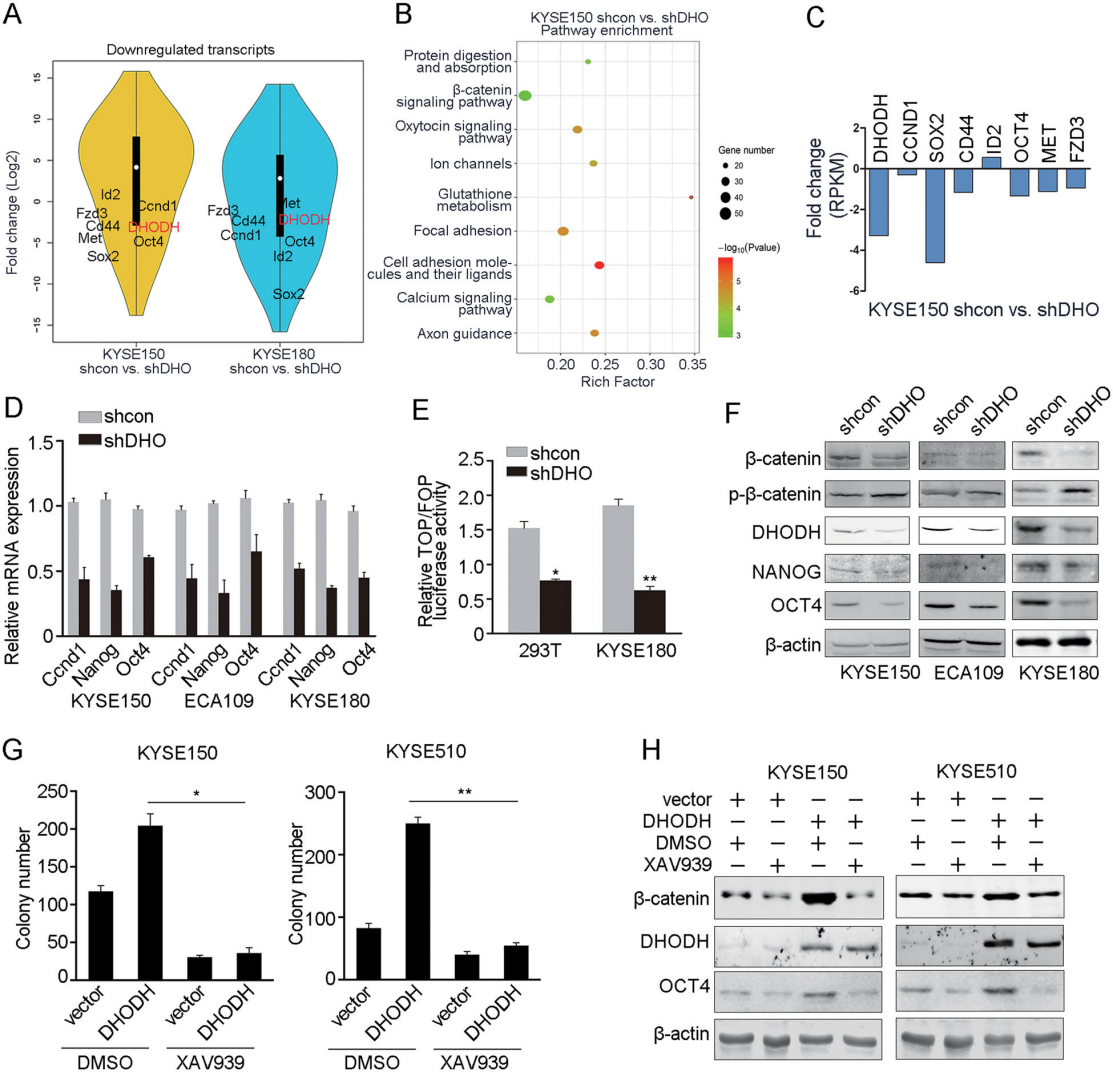
DHODH activates β-catenin signaling. **a** Comparison of gene expression pattern between DHODH knockdown (shDHO) and control cells (shcon) in KYSE150 and KYSE180 cell lines by RNA-seq, respectively. Most of β-catenin related genes as well as DHODH were downregulated. **b** Pathway enrichment analysis showed that PI3K-Akt/β-catenin pathway, protein digestion and absorption, ion channels were significantly enriched. **c-d** The expression levels of β-catenin regulated genes identified via RNA-seq (**c**) and qRT-PCR (**d**). **e** TOP/FOP flash assay depicted β-catenin downstream transcriptional activity was suppressed in DHODH knockdown 293T and KYSE180 cells. **f** Phospho-β-catenin, β-catenin, Nanog, and Oct4 were examined in DHODH knockdown and control KYSE150, KYSE180, and ECA109 cell lines, respectively. **g** Colony formation was detected after treated with XAV-939 in the control and DHODH overexpression cells. **h** XAV-939 impaired the upregulation of β-catenin and Oct4 by DHODH in KYSE510 and KYSE150 cells. Data were representative of three independent experiments and are presented as mean ± SEM. ^*^P < 0.05, ^**^P < 0.01.

Then, we performed RNA-sequencing on DHODH knockdown KYSE150, KYSE180 and ECA109 cells, respectively (Fig. 3a and Supplementary Fig. S3A). By KEGG pathway enrichment analysis, we classified top significantly altered signaling pathways that were consistent from all three cell lines. Wnt/β-catenin signaling was one of the most significantly enriched pathways (Fig. 3b and Supplementary Fig. S3B, C). We noticed that the expressions of Wnt/β-catenin target genes, such as *CCND1*, *Oct4*, and *Nanog*, were dramatically decreased after *DHODH* knockdown (Fig. 3c, d). Furthermore, we found DHODH knockdown reduced the transcriptional activity of β-catenin (Fig. 3e) using the dual luciferase reporter gene assay. Importantly, DHODH regulated β-catenin on protein expression while had no effect on the mRNA level (Fig. 3f and Supplementary Fig. S3D). As shown in Fig. 3f, knocking down of DHODH increased phospho-β-catenin (p-β-catenin) level but decreased β-catenin as well as the downstream OCT4 and Nanog.

Inhibition of β-catenin signaling by XAV-939 caused restricted cell proliferation, even when DHODH was overexpressed (Fig. 3g). The expression of Oct4 also showed similar trend (Fig. 3h). Collectively, these results indicate that DHODH may act as a critical oncogene via activating Wnt/β-catenin pathway in ESCC.

### DHODH stabilized β-catenin and promotes its nucleus translocation

**Fig. 4:**
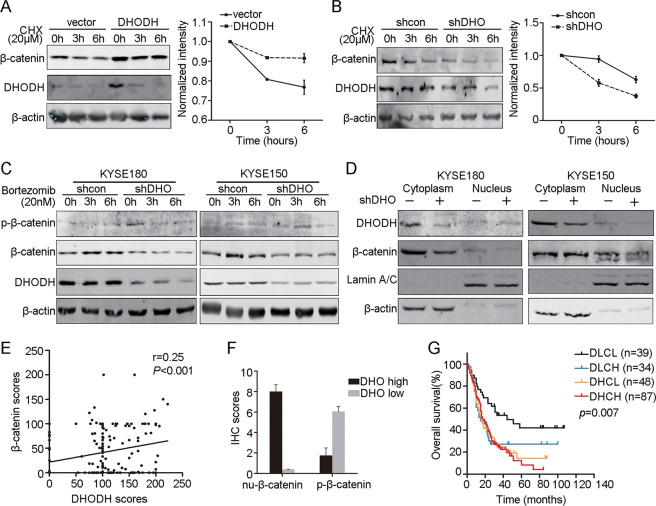
DHODH mediates β-catenin stability and nuclear localization. **a** KYSE150 cells transfected with DHODH or vector were treated with CHX for the indicated times. **b** DHODH knockdown or control KYSE180 cells were treated with CHX for the indicated times. β-catenin expression was examined by western blot (left). A plot of normalized amount of β-catenin was shown (right). **c** DHODH knockdown or control KYSE150 and KYSE180 cells were treated with proteasome inhibitor, Bortezomib, for the indicated times. **d** DHODH knockdown decreased the cytoplasm and nucleus β-catenin expression. **e** The correlation of DHODH and β-catenin expression was examined using Prism 5. **f** The expression of nuclear β-catenin and phosphor-β-catenin were examined in DHODH high cases and low cases, respectively. **g** Kaplan–Meier analysis of patient’s survival with β-catenin/DHODH co-expression (DLCL, DHODH low/β-catenin low, DLCH, DHODH low/β-catenin high, DHCL, DHODH high/β-catenin low, DHCH, DHODH high/β-catenin high). ^*^P < 0.05, ^**^P < 0.01.

As DHODH regulated β-catenin at protein level, we examined the stability of β-catenin in DHODH overexpression or knockdown cells treated with cycloheximide (CHX). We noticed that DHODH significantly stabilized β-catenin protein level (Fig. 4a, b). Consistently, the enhanced degradation of β-catenin was abrogated by bortezomib, an inhibitor of 26S proteasome complex (Fig. 4c). Moreover, DHODH knockdown reduced the nuclear localization of β-catenin (Fig. 4d).

We then investigated the relevance of DHODH and β-catenin in tumor samples. TMA analysis revealed a significant positive correlation between the expression level of DHODH and β-catenin (Fig. 4e and Supplementary Fig. S4A). Moreover, samples showing strong DHODH and β-catenin staining also exhibited strong nuclear staining of β-catenin (Fig. 4f). Moreover, patients exhibited low expression of both DHODH and β-catenin had longer overall survival than other patients (Fig. 4g).

This expression pattern was further confirmed in the slides of xenograft tumor sections derived from DHODH knockdown ECA109 cells and control cells (Supplementary Fig. S4B, C). These data suggest that DHODH was positively correlated with β-catenin at protein level in ESCC; and DHODH may involve in the regulation of β-catenin phosphorylation, thus affecting the degradation of β-catenin.

### DHODH binds the NH2 terminal of β-catenin and prevents its polyubiquitination

**Fig. 5:**
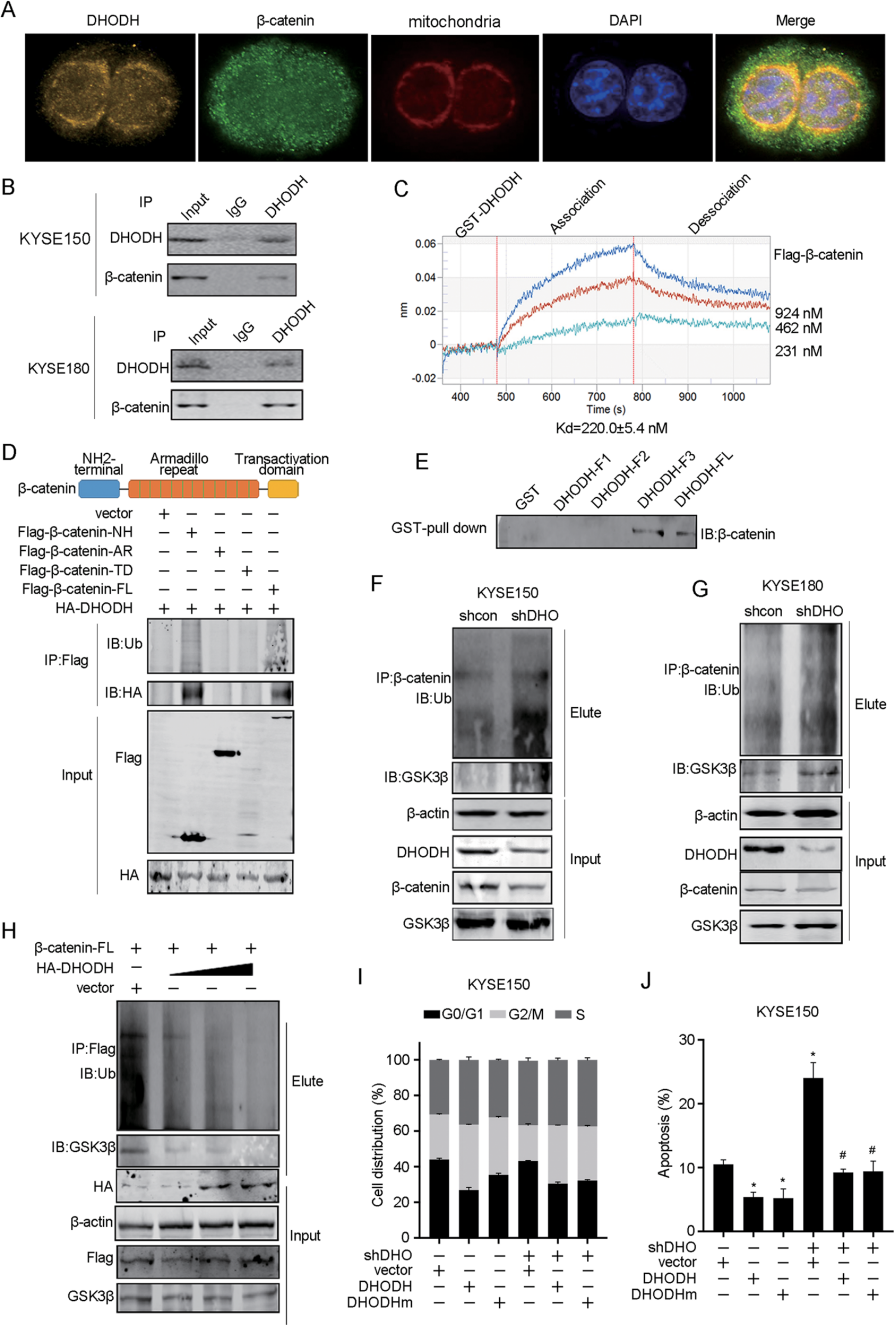
DHODH interacts with β-catenin and prevents its polyubiquitination. **a** Immunofluorescent staining of DHODH, mitochondria and β-catenin. Nuclear DNA was visualized by DAPI staining. **b** Endogenous β-catenin was immunoprecipitated with anti-DHODH antibody or control IgG and analyzed by western blot in KYSE150 and KYSE180 cells. **c** Bio-layer interferometry assay of Flag-β-catenin binding to immobilized GST-DHODH. Kinetic analysis of the affinity of β-catenin and DHODH was performed. **d** A schematic diagram of different domains of β-catenin. Each domain was co-transfected into HEK-293T cells with DHODH, and the immunoprecipitated DHODH was examined by western blot. NH, NH_2_-terminal, AR, Armadillo repeat, TD, Transactivation domain, FL, full-length. **e** GST-pull down assay was performed and the binding β-catenin was examined by western blot. **f**, **g** The polyubiquitination of β-catenin was examined in DHODH knockdown KYSE150 (**f**) and KYSE180 (**g**) cells. **h** The polyubiquitination of β-catenin was examined after transfecting DHODH in gradient amount. **i** KYSE150 knocking down of DHODH cells were transfected with DHODH wild type or DHODH functional mutation (DHODHm), respectively. Cell cycle distribution was examined. **j** Apoptosis assay of the abovementioned cells. Data were representative of three independent experiments and are presented as mean ± SEM. ^*^P < 0.05, compared to control group; ^#^*P* < 0.05, compared to shDHO alone group.

We observed that DHODH localized at not only mitochondria but also nucleus, implying its unrevealed role in addition to catalyzing dihydroorotate (Supplementary Fig. S5A). DHODH was found to be co-localized with β-catenin by IF (Fig. 5a). Furthermore, endogenous β-catenin was readily detected in DHODH immunoprecipitates (Fig. 5b). Most importantly, bio-layer interferometry binding assay showed that the purified recombinant-DHODH protein directly bound to β-catenin, forming a 1:1 complex with a dissociation constant of ~220 nM (Fig. 5c). We constructed different domains of β-catenin including the NH_2_ terminal (NH), the Armadillo repeat domain (AR), and the transactivation domain (TD). As shown in Fig. 5d, HA-tagged DHODH was observed in the Flag-β-catenin-NH and Flag-β-catenin full-length immunoprecipitates. In parallel experiments, β-catenin were specifically detected in GST-DHODH catalyze domain and full-length immunoprecipitates as revealed by GST-pull down assay (Fig. 5e and Supplementary Fig. S5B). These data suggest that the NH_2_ terminal (NH) domain of β-catenin and the catalyze domain of DHODH are both required for the interaction.

We found that the level of polyubiquitin β-catenin complex was significantly increased in DHODH knockdown cells (Fig. 5f, g), as well as co-immunoprecipitated GSK3β level. Conversely, ectopic expression of DHODH decreased the ubiquitin level of β-catenin and GSK3β level in a dose-dependent manner (Fig. 5h). Given the fact that phosphorylation by GSK3β is critical for β-catenin stability, we used GSK3β inhibitor CHIR99021 and found that inhibition of GSK3β elevated the expression of β-catenin even DHODH was knocked down (Supplementary Fig. S5C).

Furthermore, we detected the catalytic dysfunctional DHODH-mut (R135C) and found that DHODHm could restore β-catenin expression, as well as wild-type DHODH (Supplementary Fig. S5D). Also, DHODHm promoted cell growth, cell cycle and inhibited apoptosis (Fig. 5i, j and Supplementary Fig. S5E, F).

## Discussion

In this study, we found that DHODH overexpression promoted cell proliferation and tumor growth whereas DHODH knockdown caused reverse effects. However, the mechanism of DHODH mediated tumor progression was not fully investigated in ESCC.

β-catenin, first identified as a subunit of the cell to cell adhesion protein complex, acts critical role in the canonical Wnt Signaling. Targeting β-catenin activity and function could be therapeutic opportunities for many types of cancer, including ESCC^22–24^. Recently, numerous crosstalk with β-catenin were identified, including important pathways, non-coding RNAs, and metabolites^25–27^. For example, Pyk2 could decrease p-β-catenin and stabilize β-catenin, while PRL-3 activates β-catenin signaling pathway through Leo1 dephosphorylation in acute myeloid leukemia^28,29^. Canonical regulation of β-catenin linked with Wnt1 class ligand and receptors activation such as FZD and LRP5/LRP6^30^. Destruction complex containing Axin, APC, CK1α/δ, and GSK3α/β couldnot phosphate β-catenin at several Ser/Thr site, and β-catenin will translocate into nuclear. Otherwise, p-β-catenin targeted by E3-ubiquitin ligase β-TrCP is degraded by ubiquitin-proteasome pathway^31^. However, Wnt5a type ligands or Wnt/Ca^2+^ acted as the non-canonical pathways regardless of β-catenin^32,33^. In our results, GSK3β inhibitor CHIR99021 could rescue the expression of β-catenin in DHODH knocking down cells. Therefore, our hypothesis would be activation of β-catenin pathway by DHODH via the way of effectiveness on phosphorylation of β-catenin state.

As a key enzyme in de novo pyrimidine biosynthesis, DHODH catalyzes the conversion of dehydration of dihydroorotate into orotate^34^. However, in the present study, we found that upregulation of β-catenin expression was triggered by catalyzing dysfunction form of DHODH, suggesting that activating β-catenin pathway by DHODH was probably independent of its catalytic role in ESCC. Moreover, the interaction between GSK3β and β-catenin was partially abrogated after DHODH overexpression. Together, these data implied that DHODH promoted β-catenin stability by interrupting GSK3β mediated phosphorylation on the canonical pathway. Hence, inhibition of DHODH could impair β-catenin mediated tumorigenesis.

Many DHODH inhibitors have shown potential anti-cancer effects, such as ML390, brequinar, and chiral tetrahydroindazole (HZ00)^35–37^. Some of them had been approved by FDA for acute myeloid leukemia^38^. Emerging evidence suggests that inhibition of DHODH impairs cancer cell proliferation, inducing differentiation. Also, downregulation of DHODH may have synergic effects together with inhibiting other oncogenes^39–43^. Of note, PTEN-mutant cancer was sensitive to DHODH inhibition, causing significant inadequate ATR activation and DNA damage^44^. Likewise, DHODH inhibition showed strong antitumor activity in mutant-KRAS-driven cancers^45^. Targeting DHODH and disruption of the pyrimidine biosynthesis are demonstrated to be an approach to small cell lung cancer and colorectal cancer therapy^46,47^. However, the molecular mechanisms of DHODH effecting on cancer development other than pyrimidine biosynthesis remains to be elucidated. We revealed that interaction between DHODH and β-catenin contributed to the alteration of malignancy phenotypes of ESCC. This may pave a way to probe a new molecular mechanism regarding to DHODH gene in cancer cell.

The clinical significance of our investigation revealed that patients with high expression of DHODH were predominant in early stage and had a much worse prognosis than ESCC patients with lower level. Therefore, detecting the expression level of DHODH may be a potential approach to early diagnosis or predicting outcomes for ESCC patient.

## Supplementary information

Supplementary information

Supplementary figure 1

Supplementary figure 2

Supplementary figure 3

Supplementary figure 4

Supplementary figure 5

Supplementary table 1

Supplementary table 2
