# Supplementary material for: Elevated DHODH expression promotes cell proliferation via stabilizing β-catenin in esophageal squamous cell carcinoma
Source: Cell Death Dis. 2020 Oct 15;11(10):862. doi: 10.1038/s41419-020-03044-1 (PMC7566478; doi:10.1038/s41419-020-03044-1)
Supplement: Supplementary file 1 — Supplementary information [file 41419_2020_3044_MOESM1_ESM.docx]

**Supplementary Information**

Supplementary Information 2 Tables, 5 figures and figure legends.

**Supplementary figure legends**

**Supplementary Fig. S1 a** Expression pattern of DHODH in esophageal mucosa, atypical hyperplasia, carcinoma in situ, and carcinoma. **b** DHODH staining score in different stages of ESCC. **c** The protein (left) and mRNA (right) levels of DHODH were examined in various ESCC cell lines and immortal epithelial cells. **d** The mRNA and protein levels of DHODH were detected in overexpressed DHODH and control KYSE510, KYSE150 cells, respectively. **e** The mRNA and protein levels of DHODH were detected in KYSE150, KYSE180, ECA109 cells with stable knocking down of DHODH (shDHO-1 and shDHO-2) and control cells (shcon), respectively. **f** KYSE150, KYSE180, KYSE510 and ECA109 cells were treated with leflunomide (10μM) or DMSO, and cell proliferation was detected. n=3. Data were representative of three independent experiments and are presented as mean ± SEM. ^*^, *P* < 0.05, ^**^, *P* < 0.01.

**Supplementary Fig. S2** **a** DHODH knockdown decreased G2/M phase cell population whereas increased S phase cell population in KYSE150 and KYSE180 cells. **b** Cell cycle was captured using Live Cell Imaging System and the time of each cell cycle phase was measured. **c** Protein markers specific for each cell cycle were examined at different time point after cell cycle synchronization in DHODH knockdown or control KYSE150 and KYSE180 cells, respecitively. **d** DHODH knockdown increased cell apoptosis in KYSE150 (left) and KYSE180 cells (right). **e** The cell cycle distribution was analyzed in overexpressed *DHODH* and control KYSE150, KYSE510 cells, respectively. **f** The cell apoptosis rate was examined in overexpressed *DHODH* and control KYSE150, KYSE510 cells, respectively. **g** DHODH knockdown inhibited protein expressions of Bcl2, CCNE1, CCND1, and E2F3 while increased the expressions of Bax, p53, and p21. **h** Indicated proteins were detected in overexpressed *DHODH* and control KYSE150, KYSE510 cells, respectively. n=3. Data were representative of three independent experiments and are presented as mean ± SEM. ^*^, *P* < 0.05.

**Supplementary Fig. S3 a** The volcano plot of upregulated and downregulated genes in *DHODH* knockdown (shDHO) and control (shcon) ECA109 cells. **b-c** Gene enrichment pathways were showed in *DHODH* knockdown (shDHO) and control (shcon) KYSE180 (**b**) or ECA109 (**c**) cells. **d** The mRNA expression of β-catenin was examined in *DHODH* knockdown (shDHO) and control (shcon) ECA109, KYSE150, KYSE180 cells, respectively. Data were representative of three independent experiments and are presented as mean ± SEM. ^**^, *P*<0.01.

**Supplementary Fig S4** **a** Representative images showed the expression of β-catenin and phosphor-β-catenin in 208 cases of ESCC tissues (Bar, 100μm). **b** The staining intensities were measured by ImageJ software in pictures taking from IHC staining of DHODH, β-catenin on tissue slides of tumors bearing *DHODH* knockdown (shDHO) or control (shcon) cells. **c** IHC scores of β-catenin and phosphor-β-catenin were detected by Aperio Scan Scope. ^*^, *P* < 0.05.

**Supplementary Fig S5** **a** Immunofluorescence staining in KYSE150 cells. Cells were indicated by DHODH (Green), mitochondria (Red), and DAPI (blue). **b** A schematic diagram of different domains of GST tagged DHODH. **c** KYSE150 and KYSE180 cells knocking down of DHODH and control cells were treated with GSK3β inhibitor (CHIR99021, 5μM) and indicated proteins were examined by western blot. **d** KYSE150 and KYSE180 cells knocking down of DHODH were transfected with DHODH wild type or DHODH functional mutation (DHODHm), respectively. Indicated proteins were examined. **d** Cell cycle distribution of the abovementioned KYSE180 cells. **e** Apoptosis assay of the abovementioned KYSE180 cells. Data were representative of three independent experiments and are presented as mean ± SEM. ^*^ , *P* < 0.05, ^**^, *P*<0.01, compared to control group; ^##^, *P*<0.01, compared to shDHO alone group.
