# Supplementary figures and images for: Elevated DHODH expression promotes cell proliferation via stabilizing β-catenin in esophageal squamous cell carcinoma
Source: Cell Death Dis. 2020 Oct 15;11(10):862. doi: 10.1038/s41419-020-03044-1 (PMC7566478; doi:10.1038/s41419-020-03044-1)

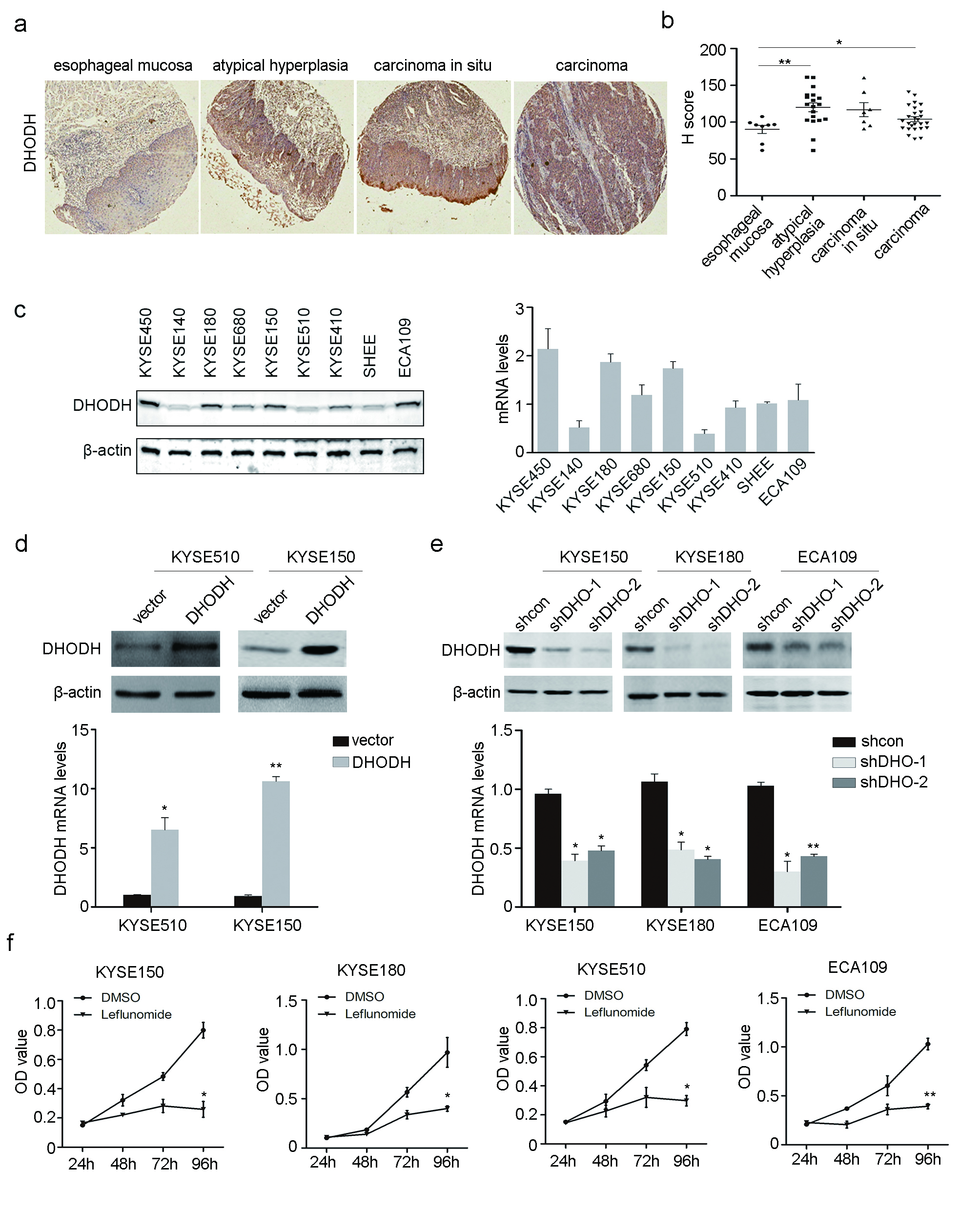

Supplement: Supplementary file 2 — Supplementary figure 1 [file 41419_2020_3044_MOESM2_ESM.jpg]

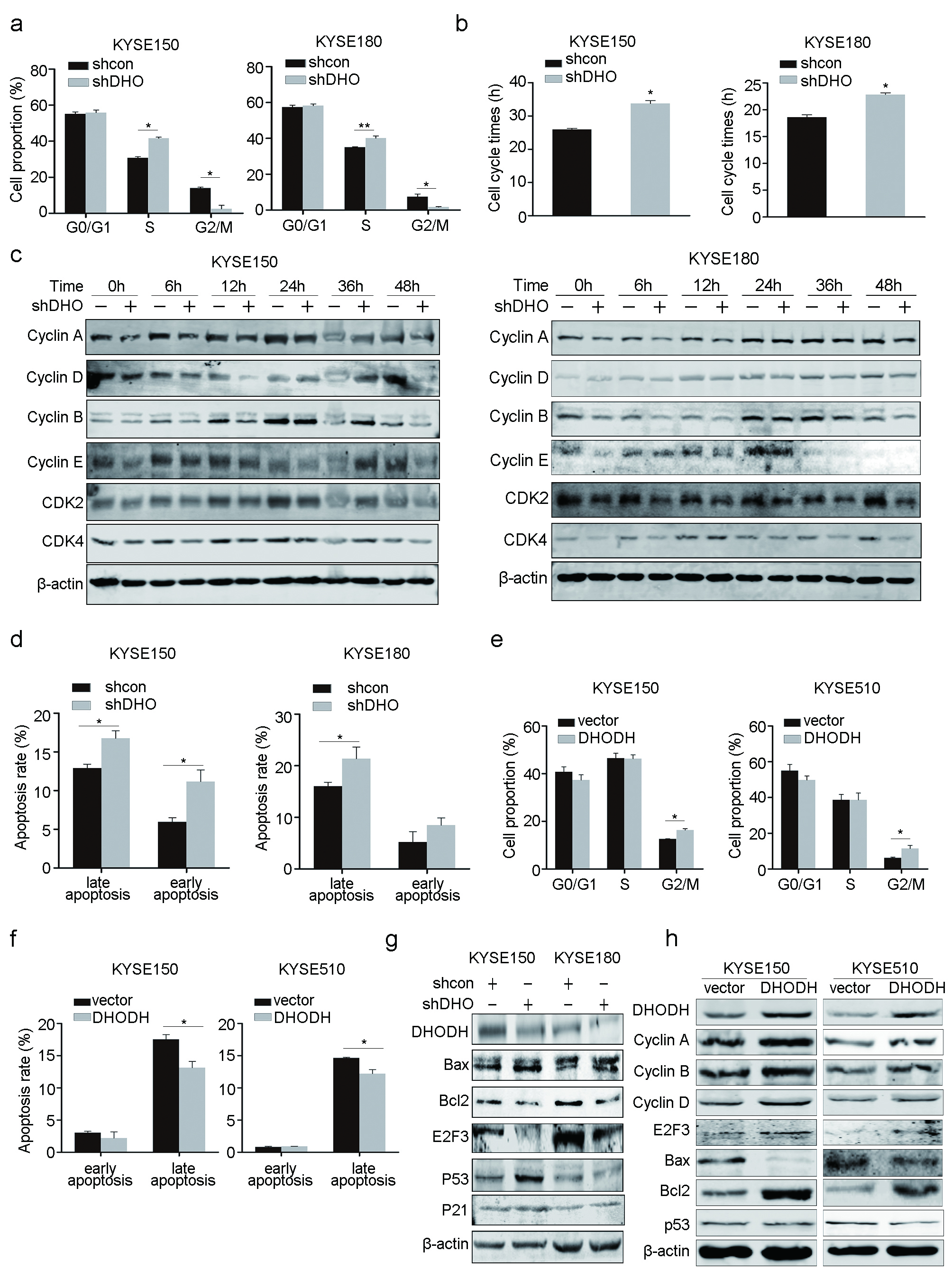

Supplement: Supplementary file 3 — Supplementary figure 2 [file 41419_2020_3044_MOESM3_ESM.jpg]

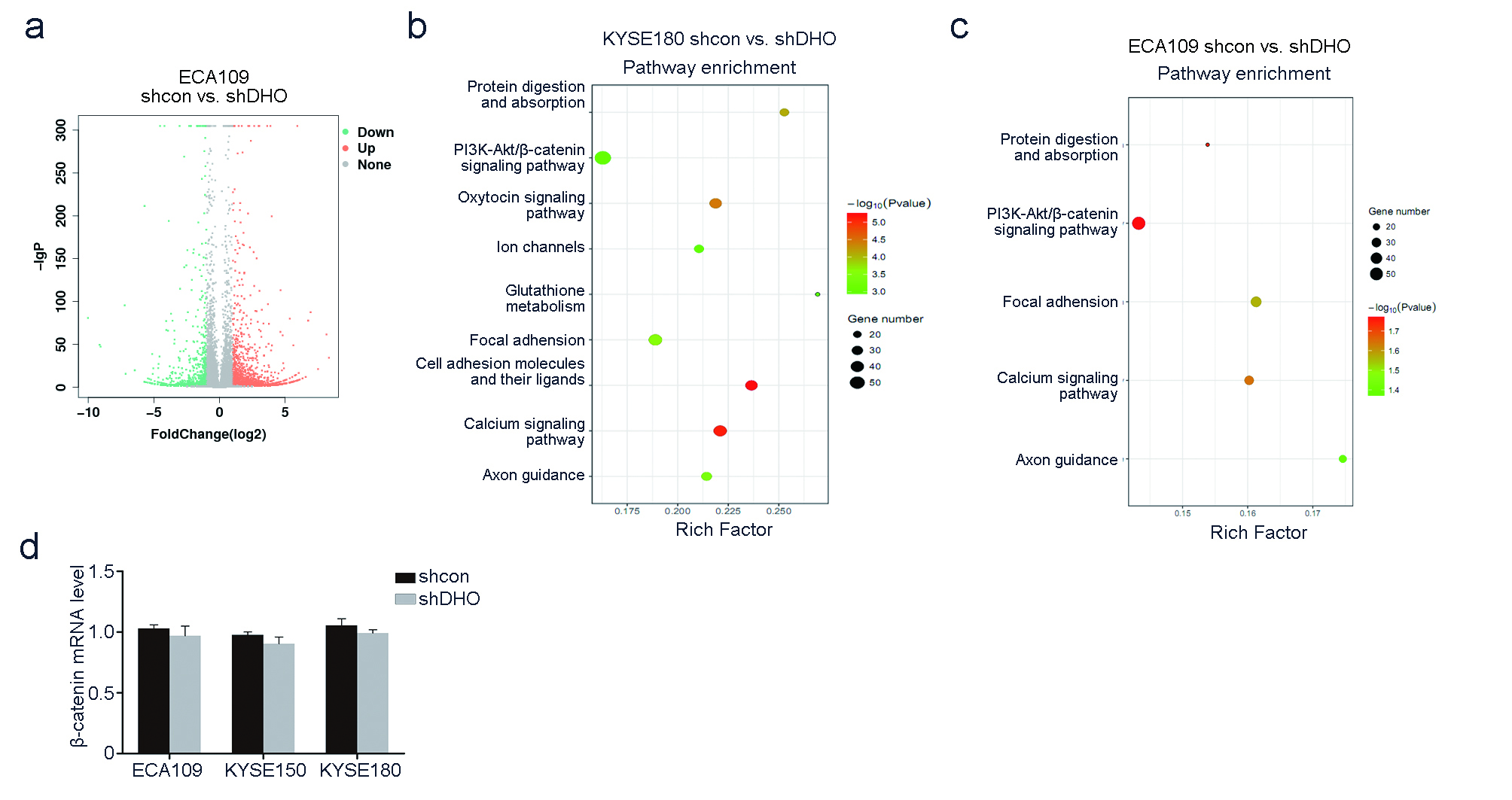

Supplement: Supplementary file 4 — Supplementary figure 3 [file 41419_2020_3044_MOESM4_ESM.jpg]

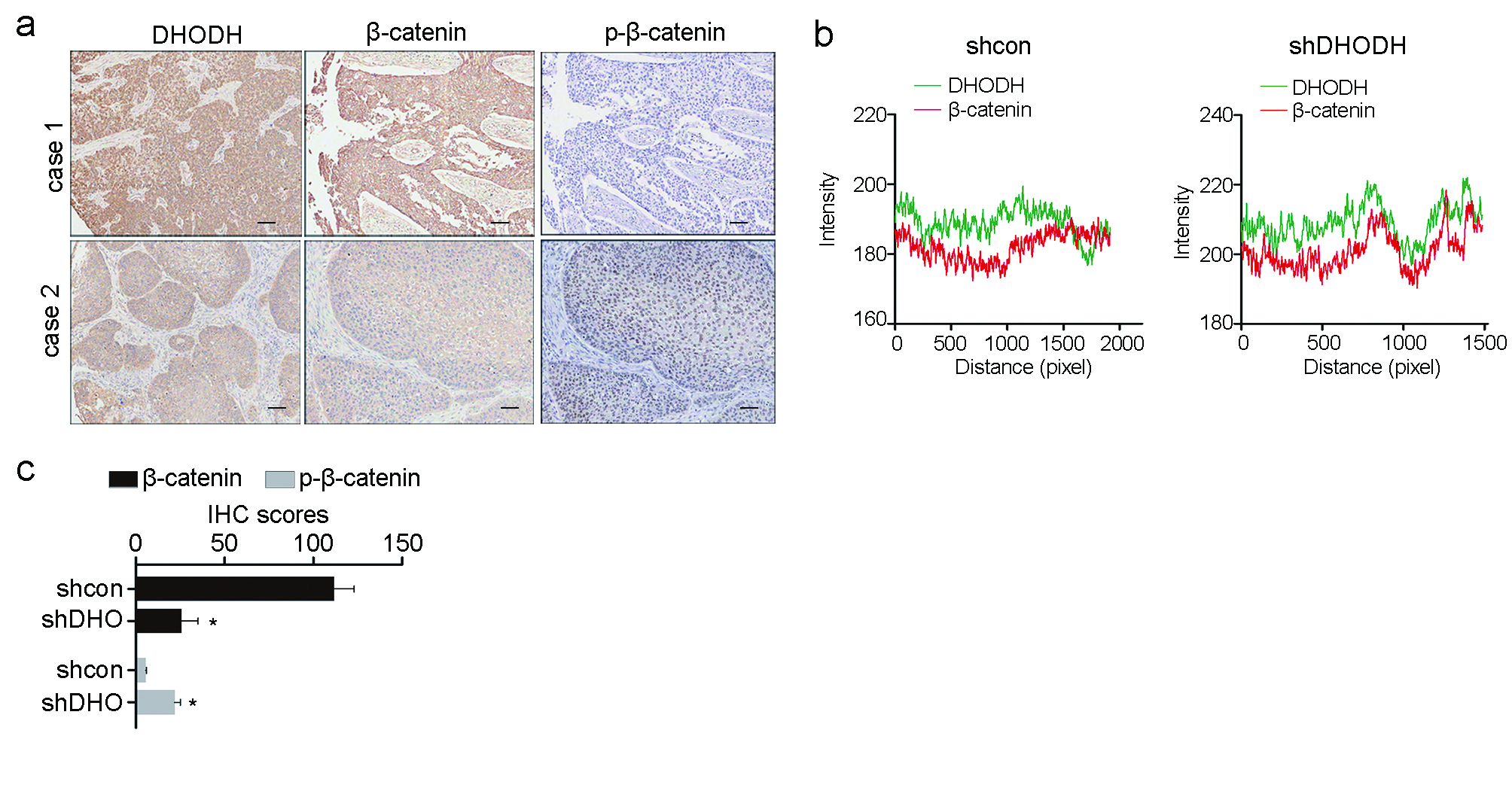

Supplement: Supplementary file 5 — Supplementary figure 4 [file 41419_2020_3044_MOESM5_ESM.jpg]

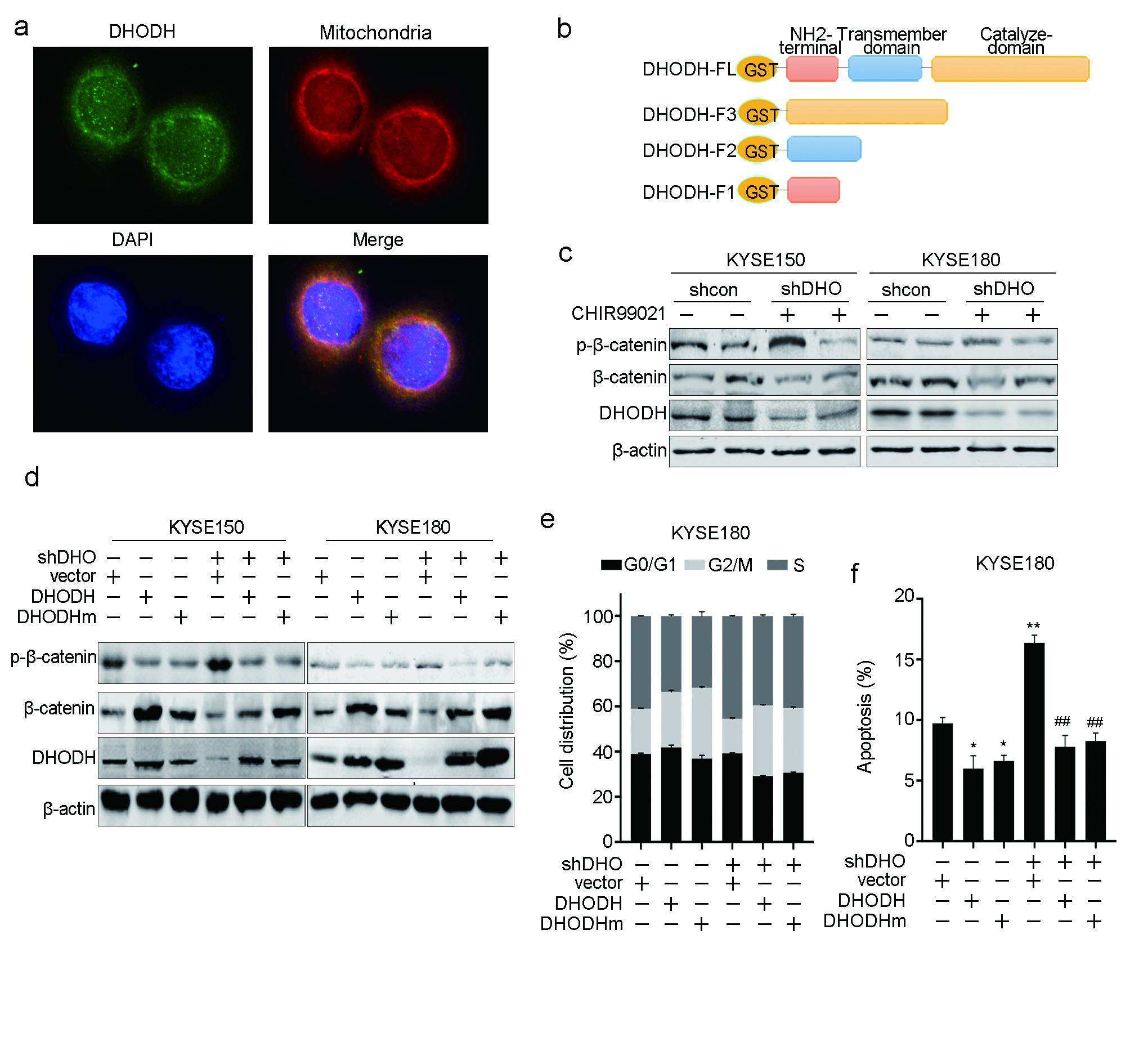

Supplement: Supplementary file 6 — Supplementary figure 5 [file 41419_2020_3044_MOESM6_ESM.jpg]
