# Supplementary material for: Elevated DHODH expression promotes cell proliferation via stabilizing β-catenin in esophageal squamous cell carcinoma
Source: Cell Death Dis. 2020 Oct 15;11(10):862. doi: 10.1038/s41419-020-03044-1 (PMC7566478; doi:10.1038/s41419-020-03044-1)
Supplement: Supplementary file 8 — Supplementary table 2 [file 41419_2020_3044_MOESM8_ESM.pdf]

Supplementary Table 2 Primers

For PCR

Genbank Accession	Gene name	Forward primer (5’-3’)	Reverse primer (5’-3’)
NM_001361	DHODH	CGGGATCCatggcgtggagacacctgaaaaag	GCTCTAGAtcacctccgatgatctgctccaatg

For qRT-PCR

Genbank Accession	Gene name	Forward primer (5’-3’)	Reverse primer (5’-3’)
NM_001361	DHODH	CCACGGGAGATGAGCGTTTC	CAGGGAGGTGAAGCGAACA
NM_001101	β-actin	GCCGCCAGCTCACCAT	CACGATGGAGGGGAAGACG
NM_053056	Ccnd1	GCTGCGAAGTGGAAACCATC	CCTCCTTCTGCACACATTTGAA
NM_024865	Nanog	GTCCCGGTCAAGAAACAGAA	TGCGTCACACCATTGCTATT
NM_002701	Oct4	ATTCAGCCAAACGACCATCT	ACACTCGGACCACATCCTTC
NM_001949	E2f3	AGAAAGCGGTCATCAGTACCT	TGGACTTCGTAGTGCAGCTCT
NM_001904	β-catenin	CATCTACACAGTTTGATGCTGCT	GCAGTTTTGTCAGTTCAGGGA
NM_000633	BCL2	GGTGGGGTCATGTGTGTGG	CGGTTCAGGTACTCAGTCATCC
